# Health-Related Quality of Life in Relation to Health Behaviour Patterns among Canadian Children

**DOI:** 10.3390/children11030346

**Published:** 2024-03-14

**Authors:** Xiuyun Wu, Arto Ohinmaa, Paul J. Veugelers, Katerina Maximova

**Affiliations:** 1 School of Public Health, University of Alberta, Edmonton, AB T6G 1C9, Canada; 2 MAP Centre for Urban Health Solutions, Li Ka Shing Knowledge Institute, St. Michael’s Hospital, Toronto, ON M5B 1T8, Canada; 3 Dalla Lana School of Public Health, University of Toronto, Toronto, ON M5T 3M7, Canada

**Keywords:** children, health-related quality of life, health behaviours, sedentary activity, physical activity, sleep, dietary intake, latent class analysis, Canada

## Abstract

Poor health behaviours in childhood, including sedentary behaviour, low physical activity levels, inadequate sleep, and unhealthy diet, are established risk factors for both chronic diseases and mental illness. Scant studies have examined the importance of such health behaviour patterns for health-related quality of life (HRQoL). This study aimed to examine the association of health behaviour patterns with HRQoL among Canadian children. Data from 2866 grade five students were collected through a provincially representative school-based survey of the 2014 Raising Healthy Eating and Active Living Kids in Alberta study. Latent class analysis was used to identify health behaviour patterns based on 11 lifestyle behaviours: sedentary behaviour (using a computer, playing video games, watching TV), physical activity (with and without a coach), sleep (bedtime on weekdays and weekends), and diet (fruit and vegetables intake, grain products, milk and alternatives, meat and alternatives). Multivariable multilevel logistic regression was applied to examine the associations of health behaviour patterns with HRQoL. Three groupings with distinct health behaviour patterns were identified: the first grouping (55%) is characterized by relatively healthy levels of sedentary behaviour, physical activity, and sleep, but a less healthy diet (“activity-focused” group). The second grouping (24%) is characterized by a relatively healthy diet, but moderately healthy levels of sedentary behaviour, physical activity, and sleep (“diet-focused” group). The third grouping (21%) is characterized by mostly unhealthy behaviours (“not health-focused” group). Students in the third and second groupings (“not health-focused” and “diet-focused”) were more likely to report lower HRQoL relative to students in the first grouping (“activity-focused”). The findings suggest that health promotion strategies may be more effective when considering the patterns of health behaviours as distinct targets in the efforts to improve HRQoL. Future research should include prospective observational and intervention studies to further elucidate the relationship between health behaviour patterns and HRQoL among children.

## 1. Introduction

Poor health behaviours in childhood, including sedentary behaviours, low physical activity levels, unhealthy eating, and inadequate sleep, have been shown to affect both chronic diseases (e.g., obesity, type 2 diabetes, and cardiovascular disease) [1,2,3], and mental health (e.g., depression, anxiety, and low psychosocial well-being) [4,5,6]. Poor health behaviours in childhood have also been shown to be associated with health-related quality of life (HRQoL), a measure of self-perceived health covering a multidimensional construct of physical and psychological health and well-being, and social functioning [7,8]. Systematic reviews have revealed that children and adolescents with sedentary lifestyles, low levels of physical activity, and/or unhealthy diets are more likely to report poor HRQoL compared to their peers with healthier behaviours [7,8]. Research among children and adolescents has also revealed that insufficient sleep is related to poor HRQoL [9].

Health behaviours often co-occur in patterns that may induce synergistic and cumulative effects on health [10]. Various studies among children have revealed associations of health behaviour patterns or clusters with health outcomes including obesity [10,11] and mental health [12], as well as socio-economic factors [13,14]. Research has also revealed that that behaviour patterns characterized by low PA, high sedentary behaviour, short sleep time, and low consumption of fruit and vegetables, grain products, and high consumption of fatty foods, such as snack foods, were linked with overweight/obesity in youth [10,15]. Moreover, some studies reported that unhealthy behaviour patterns were associated with higher odds of anxiety, depression, or suicidal behaviours [12,16]. Although the associations between single or individual health behaviours and HRQoL among children and youth have been extensively examined, scant studies have investigated how the health behaviour patterns or otherwise combinations of the aforestated health behaviours are related to HRQoL. We have found only three studies [17,18,19] that examined combinations or patterns of health behaviours in relation to HRQoL in childhood and adolescence, and none of them consider sleep, which is known to be an important determinant of HRQoL [9,20]. In addition, few studies considered the various dietary aspects or food groups in relation to HRQoL [7,8]. To our best knowledge, no studies among children have reported patterns of health behaviours that considered simultaneously physical activity, sedentary behaviour, diet and sleep, in association with HRQoL. Given that HRQoL comprises multiple aspects of health (e.g., physical and psychosocial health), a better understanding of the impact of health behaviour patterns on HRQoL may guide the tailoring of health promotion interventions for children to promote healthy lifestyles and prevent chronic disease and mental illness.

Cluster analysis and latent class analysis (LCA) are two major data-driven, person-centered methods to investigate co-occurring patterns or clusters of health-related behaviours among various populations [10,21]. Previous studies using the individual-centered statistical method mostly applied traditional cluster analysis [10,21]. In the past decade, LCA has been used to assess a wide range of health-related behaviours, such as physical activity, sedentary behaviour, diet or eating habits, alcohol consumption, tobacco use, and other behaviours [10,11,15,22]. LCA is a multivariate statistical modeling method that aims to identify unobserved or latent groups of individuals with distinct response patterns on multiple item indicators within a population [23]. Relative to conventional cluster analysis, one advantage of the LCA is that it is based on statistical modeling that provides a set of fit statistics, which facilitate the selection of the most suitable model [24]. Further, a latent class regression model can simultaneously include covariates and/or a distal outcome to assess their associations with the latent class membership [23]. LCA is particularly appropriate for analyzing different types of categorical behavioural variables, which are frequently used in health behaviour assessments.

In this study, we sought to identify patterns of health behaviours using a comprehensive range of health behaviour indicators, and to examine the associations of the health behaviour patterns with HRQoL among children. We also sought to examine correlations of the health behaviour patterns with socio-demographic characteristics, body weight status, and overall diet quality in children. We used LCA to identify subgroups of children with distinct response patterns on health behaviours.

We hypothesized that homogeneous subgroups of children would be identified in a meaningful manner and that a subgroup with an elevated unhealthy level of behaviour pattern would have a higher likelihood of lower HRQoL. We also hypothesized that the behaviour patterns would be associated with body weight status, overall diet quality, and some socio-economic status among children.

## 2. Methods

### 2.1. Study Procedures and Participants

Data were drawn from a provincially representative sample of children collected through the 2014 Raising Healthy Eating and Active Living Kids in Alberta (REAL Kids Alberta) survey, which aimed to evaluate government health promotion efforts. The school-based survey was conducted in Spring 2014 among grade five students who are primarily 10–11 years of age, and among their parents. The surveys applied a one-stage stratified random sampling design among 90.2% of the elementary schools in Alberta, Canada (francophone schools, on-reserve federal schools, and private, charter, and colony schools were excluded and comprised the remaining 9.8% of schools in the province). From three geographic regions (cities with population ≥ 100,000; towns with population ≥ 40,000; and rural (municipalities with population < 40,000), we randomly selected 140 schools. A total of 2958 students received parents’ consent and participated in this study (participation rate = 59%). After further excluding students who provided incomplete information on the health behaviours (*n* = 92), 2866 students were available for analysis. Details on the design and data collection procedures have been reported elsewhere [25].

Students at school completed an in-class questionnaire comprising questions on physical activity, screen-based sedentary behaviours, and sleep time. The survey also comprised the Harvard Youth/Adolescent Food Frequency Questionnaire (YAQ) [26,27] adapted for use in Canada, and the EQ-5D-Y questionnaire to assess health-related quality of life [28]. Parents completed information on children’s socio-demographic characteristics, including gender, region of residence, parental education, and household income. Student’s standing height was measured by trained research assistants to the nearest 0.1 cm using stadiometers (Seca-Stadiometers, Hamburg, Germany) and student’s weight was measured to the nearest 0.1 kg using calibrated digital scales (Health-o-meter^®^, Alsip, IL, USA). Parental consent and student assent for participation were obtained before the administration of the survey. All study procedures and analyses received approval from the Health Research Ethics Board of the University of Alberta.

### 2.2. Measures

#### 2.2.1. Health-Related Quality of Life

The HRQoL was assessed by the EQ-5D-Y (youth) [28] that consists of five dimensions: (a) walking; (b) looking after myself; (c) doing usual activities; (d) having pain or discomfort; and (e) feeling worried, sad, or unhappy. Each of these dimensions has three response levels: no problems, some problems, or a lot of problems. The second part of the instrument is a Visual Analogue Scale (VAS) with a score ranging between “100” representing best imaginable health and “0” representing worst imaginable health, asking participants to self-report their overall health status. The EQ-5D-Y has been validated and is available in many languages around the world [29] (https://euroqol.org/eq-5d-instruments/eq-5d-y-about, accessed on 7 March 2024). This study used the five-dimensional descriptive system of the EQ-5D-Y. The reliability coefficient (Cronbach’s alpha) of the EQ-5D-Y in this study was 0.61.

#### 2.2.2. Physical Activity, Sedentary Behaviour, and Sleep Time

Two questions queried students on their participation in organized sports or physical activity with and without a coach, adopted from the National Longitudinal Survey for Children and Youth in Canada [30]. Students reported their frequency of participation in physical activity as never, 1–3 times a week, and ≥4 times a week. Students were asked to report the number of hours and minutes spent on using computers, playing video games, and watching television (TV). The response options were categorized into three levels: <1 h/day, 1–2 h/day, or ≥3 h/day. Parents were asked to report the time their children went to bed on school days and on weekends, with the following response options: before 9 p.m., 9–10 p.m., and after 10 p.m.

#### 2.2.3. Dietary Intake and Diet Quality

The YAQ contains questions on dietary consumption of various food items (e.g., milk, meat, vegetables, and fruit). Nutrient intakes were calculated based on student responses to these questions, using the Canadian Nutrient File [31]. We included the following dietary measures: (1) the number of daily servings of the four major food groups: vegetables and fruit, grain products, milk and alternatives, and meat and alternatives; and (2) whether students met the number of daily servings recommended for their age group [32]. Specifically, the recommendations are as follows: ≥5 servings per day for vegetables and fruit; ≥5 servings per day for grain products; ≥3 servings per day for milk and alternatives; and ≥2 servings per day for meat and alternatives. The diet quality was quantified using the Diet Quality Index-International (DQI-I) [33], which considers both nutrient and food intake [26]. The DQI-I scores range from 0 to 100, whereby higher scores indicate better diet quality. We considered the DQI-I scores as tertiles.

#### 2.2.4. Socio-Demographic Characteristics and Body Weight

Socio-demographic characteristics included student’s gender, region of residence, parental education, and household income. Students reported their gender (girl, boy). Region of residence was classified as urban, which included medium and large population centres 40,000–99,999 and ≥100,000, respectively, and rural, which included small population centres < 40,000. The highest level of parental education was categorized as secondary school or less, college, and university. Household income categories included CAD < 50,000, CAD 50,001–75,000, CAD 75,001–100,000, and CAD > 100,000 per year. We used the International Obesity Task Force age- and gender-specific body mass index (BMI) cut-off points for children to create weight status categories of normal weight, overweight, and obese [34].

### 2.3. Statistical Analysis

We applied latent class analysis to complex survey data to identify groupings (latent classes) of students with different patterns of health behaviours. The items used in the LCA to identify health behaviour patterns included the 11 aforementioned health behaviours (three items for sedentary behaviour, two items for physical activity, two items for sleep, and four items for diet). All items were categorical and coded such that a higher score was indicative of less healthy behaviour. This coding of the health behaviours is detailed in the Supplementary Materials (Table S1). Previous studies among children and youth with distinct item response patterns had demonstrated that heterogeneous latent groupings (classes) can be identified when using categorical items [9]. We estimated successive LCA models ranging from a one-class model to a four-class model. The latent class model fit was assessed using the Bayesian Information Criterion (BIC), the sample size-adjusted BIC (aBIC), and the Lo–Mendell–Rubin adjusted likelihood ratio test (LMRALRT) [35]. A lower BIC and aBIC for a *k* class model indicate a better fit of this *k* class model compared to the *k* − 1 class model [35]. A *k* class model with a *p*-value > 0.05 by the LMRALRT indicates a better fit of the *k* − 1 class model compared to the *k* class model.

We adopted a manual stepwise approach in the LCA and in the examination of associations of the groupings (latent classes) with HRQoL and other explanatory variables [23,36]. We estimated the latent class model with 11 health behaviour variables using maximum likelihood estimation with robust standard errors (MLR) [23]. Once the final latent class model with optimal number of groupings (latent classes) was selected, the predicted class for each observation was then obtained representing the health behaviour pattern. The class membership groupings (classes) can be explained by patterns of probabilities across the health behaviour items (indicators). Subsequently, we examined the associations of these health behaviour patterns with HRQoL, socio-demographic characteristics (gender, region of residence, parental education, and household income), body weight status, and diet quality, using a series of logistic regression models. We applied multilevel multinomial logistic regression models to examine the relationships of the health behaviour patterns (represented by latent classes) with socio-demographic characteristics, body weight status, and diet quality, respectively. We applied multilevel multivariable logistic regression models to assess the relationship of health behaviour patterns (latent classes) with each of the dimensions of the EQ-5D-Y while adjusting for the potential confounding influence of the socio-demographic characteristics. We dichotomized the responses of each of the EQ-5D-Y dimensions by combining “some problems” and “a lot of problems” into one level. We considered missing values for parental education and household income as separate covariate categories (estimates not presented). All analyses including LCA were weighted to ensure representativeness of the provincial population of grade five students. Mplus version 8 was used to perform latent class analyses [23], and regression analyses were carried out using Stata/IC 15 (StataCorp LLC, College Station, TX, USA).

## 3. Results

### 3.1. Student Characteristics and HRQoL

Table 1 shows students’ socio-demographic characteristics. Overall, 52.9% were girls, 39.6% resided in a rural area, and 20.6% and 7.9% were overweight and obese, respectively. The prevalence of having some/a lot of problems on the EQ-5D-Y dimensions were 13.2%, 16.2%, 15.5%, 44.8%, and 29.2% for “walking”, “looking after myself”, “doing usual activities”, “having pain or discomfort”, and “feeling worried, sad or unhappy”, respectively.

### 3.2. Health Behaviour Patterns

The fit statistics of the latent class analysis for deriving the health behaviour patterns are presented in Table S2 (see Supplementary Materials). The Bayesian Information Criterion and the sample size-adjusted BIC decreased from one- to four-class models. The LMRALRT *p*-value for the four-class model was not statistically significant (*p* = 0.3226). However, for the three-class model, the *p*-value was 0.0096 and hence, this three-class model was selected as the most parsimonious model. The class membership was assigned for each student observation based on their most likely latent class membership.

Table 2 presents the frequency distribution of students for each of the health behaviours within the three groupings (latest classes), respectively, and overall. Students in the first group account for 55% of respondents. These students reported less sedentary time (computer, TV, and video games), higher levels of physical activity with a coach and intermediate levels of physical activity without a coach, and had longer sleep times than students in groups 2 and 3. Students in group 1 also reported lower intakes of vegetables and fruit, grain products, milk, and meat relative to students in group 2 (Table 2). The health behaviour pattern of this first group can be summarized as “low levels of sedentary behaviour, high levels of physical activity, longer sleep time, and low intakes of the four food groups” and thus termed “activity-focused”.

The second group represents 24% of students. Students in group 2 had intermediate levels of sedentary behaviour and sleep time (on school days and weekends) between groups 1 and 3. Percentages of students who reported screen time ≥ 3 h/day and bedtime after 10 p.m. are higher than group 1 but lower than group 3. The proportion of students engaged in physical activity ≥ 4 times per week in this group (45.85%) was greater than group 1 (37.56%) and group 3 (17.58%). PA with a coach in this group showed an intermediate level: more students (35.33%) reported never doing PA with a coach, which is higher than group 1 (27.24%), and lower than group 3 (50.69%). The intakes of vegetables and fruit, grains, milk, and meat in this group were highest compared to other groups. Group 2 is summarized as “healthy dietary intake with intermediate levels of sedentary behaviour, physical activity, and sleep” and termed “diet-focused”.

The third group represents 21% of students. Students in this group reported worse levels of sedentary behaviour, physical activity, and sleep time, and low intakes of vegetables, fruit, and milk relative to students in groups 1 and 2 (Table 2). Group 3 is summarized as “high levels of sedentary behaviour, low levels of physical activity, short sleep time, and poor dietary intake” and constitutes “not health-focused” (unhealthy pattern).

The mean number of daily servings and the standard deviation of vegetables and fruit, grain products, milk and alternatives, and meat and alternatives, and diet quality index score (DQI-I) are presented in Table S5.

### 3.3. Associations of Health Behaviour Patterns with HRQoL

Table 3 presents the associations of health behaviour patterns with HRQoL. Students in group 3 (“not health-focused”) were more likely to report problems on all five EQ-5D-Y dimensions, relative to those in group 1 (“activity-focused”). Students in group 2 (“diet-focused”) were more likely to report problems “doing usual activities”, “having pain or discomfort” and “feeling worried, sad or unhappy”, compared to those in group 1 (“activity-focused”). Relative to boys, girls were more likely to report problems on four dimensions of EQ-5D-Y, except for the “walking” dimension. Students residing in rural areas were more likely to report problems “feeling worried, sad or unhappy”. Students from lower income households were more likely to report problems on four of the five EQ-5D-Y dimensions (Table 3).

### 3.4. Health Behaviour Patterns According to Socio-Demographic Characteristics, Body Weight Status and Diet Quality

Table 4 presents the likelihood of group membership for the three health behaviour patterns according to socio-demographic characteristics, body weight status, and diet quality. More boys were found in group 3 (“not health-focused”) and group 2 (“diet-focused”) compared to girls. Students residing in rural areas (relative to urban areas) and from households with an annual income above CAD 75,000 (relative to CAD < 50,000) were less likely to be in group 3 (“not health-focused”). Students with parents with higher education were less likely to be in groups 2 and 3. Students who were obese were more likely to be in group 3 (“not health-focused”); and overweight students were more likely to be in group 2 (“diet-focused”). Students with better diet quality (middle and highest tertiles versus lowest tertile of DQI-I) were more likely to be in group 2 (“diet-focused”). And those with the highest diet quality (highest tertile) were less likely to be in group 3 (“not health-focused”).

The multivariable logistic regression revealed that unhealthy behaviour patterns are associated with being overweight and obese (Table S3, Supplementary Materials). The frequency distributions for each of the three groupings (latent classes) according to socio-demographic characteristics, body weight, diet quality, and EQ-5D-Y dimensions are presented in Table S4 (see Supplementary Materials).

## 4. Discussion

In this study, we identified three groupings of students with distinct health behaviour patterns. One grouping (“activity-focused”) exhibited a relatively healthy pattern of sedentary behaviour, physical activity, and sleep, but an unhealthy pattern with respect to dietary intake. Another grouping (“diet-focused”) showed a relatively healthy dietary pattern but less healthy patterns for sedentary behaviour, physical activity, and sleep. A third grouping (“not health-focused”) reported unhealthy behaviours with respect to sedentary behaviour, physical activity, sleep, and intakes of vegetables and fruit, and milk and alternatives. The present study revealed that compared to “activity-focused”, “not health-focused” reported significantly more problems on all five EQ-5D-Y dimensions whereas “diet-focused” reported problems on some dimensions. “Not health-focused” were also more likely to be from socio-economically disadvantaged households, to have poor diet quality, and to be overweight or obese.

To our knowledge, this study is the first study to examine associations between modifiable health behaviour patterns and HRQoL among school-age children using the LCA approach. Previous research on HRQoL in children and youth mainly evaluated individual lifestyle behaviours [7,8]. Healthy, unhealthy, and mixed patterns of health behaviours comprising physical activity, sedentary behaviour, diet, and sleep have been previously investigated, and their associations with health outcomes (e.g., obesity, adiposity, mental health) among children and youth have been reported [12,37,38,39]. Of these studies that examined clustering patterns of health behaviours, most utilized traditional cluster analysis. Very few studies used a regression model-based LCA approach. In contrast, studies that have examined how clustering patterns of these behaviours impact multidimensional HRQoL among children and youth are scant [17]. One study using latent profile analysis among a small sample of 204 elementary school students to examine profiles of physical activity and sedentary behaviour and their associations with HRQoL found that children in the active profile had significantly higher psychosocial HRQoL than children in inactive and moderate profiles, while they had no significant differences in physical HRQOL [19]. Another study analyzed associations of health behaviour clustering with HRQoL in a large sample of children (*n* = 5759) from 12 countries using cluster analysis, and reported that a cluster characterized by little screen time, healthy eating, and moderate PA exhibited better HRQoL than sitters [17]. We recently examined and found that eating behaviour patterns were associated with HRQoL among Canadian children aged 10–11 years [40]. The present study builds on this evidence, and further investigates the influence of health behaviour patterns on HRQoL among Canadian children. Particularly, we examined how the core components of diet intake (vegetables and fruit, grain products, meat and milk, and alternatives) co-occurred with other common health-related behaviours (sedentary behaviour, physical activity, and sleep), and how the behaviour patterns are associated with HRQoL among children [4,41].

The finding that children who are “not health-focused” (group 3) and “diet-focused” (group 2) were more likely to have lower HRQoL relative to “activity-focused” (group 1) is in line with other studies showing that children with multiple co-occurring poor behaviours had lower HRQoL [17,19]. Notably, the finding that “not health-focused” (group 3) and “diet-focused” (group 2) patterns are related to more health problems on the dimension of “feeling worried, sad or unhappy” is consistent with other studies reporting that unhealthy lifestyle behaviour patterns are associated with worse mental health [4,12]. The observed associations of health behaviour patterns of “not health-focused” (group 3) and “diet-focused” (group 2) with lower HRQoL on other EQ-5D-Y dimensions (e.g., walking, looking after myself, doing usual activities, and having pain or discomfort) are consistent with previous studies showing that poor behaviour patterns correlate with some physical health outcomes such as physical QoL, cardiorespiratory health, overweight and obesity [10,17,18,42]. Furthermore, we observed a dose-response association in the likelihood of lower HRQoL across the three health behaviour patterns: children who engaged in unhealthy lifestyle behaviours (“not health-focused”) were more likely to have worse HRQoL than those who followed healthier lifestyles (“diet-focused” and “activity-focused”), with children who had the best HRQoL being those who had healthiest lifestyles with respect to movement and sleep (“activity-focused”) (Table 3).

The association of health behaviour patterns with body weight is also consistent with previous studies [10,15]. We found that children who engaged in unhealthy lifestyle behaviour (“not health-focused”) were more likely to have overweight and obesity (Table S3), which supports the previous research findings [10,11,15]. The co-occurrence of unhealthy behaviours, such as low physical activity, excessive screen time, insufficient sleep, and inadequate dietary intake, may explain the higher prevalence of overweight and obesity among children in this group. The associations of health behaviour patterns with socio-demographic characteristics observed in the present study also support the prior research that lifestyle behaviours vary across socio-economic status, with families from lower socio-economic backgrounds bearing the brunt of poor health behaviour patterns [10,14]. In addition, the group of “diet-focused” children had better diet quality than the other groups (“not health-focused”, “activity-focused”), which also supports the hypothesis.

Interestingly, we observed that children had primarily two distinct patterns with respect to dietary intake: low quality diet (“activity-focused” and “not health-focused”) and high-quality diet (“diet-focused”). In contrast, the patterns for physical activity, sedentary behaviour, and sleep were more heterogeneous. Previous studies also found that children who met the lifestyle behaviour recommendations for specific behaviours (e.g., diet) may not necessarily meet the recommendations on other behaviours (e.g., sedentary behaviour) [11]. Our results suggest that health behaviours tend to co-occur in several different but meaningful patterns, and these patterns correlate with both physical and mental health outcomes such as HRQoL and overweight and obesity in children and youth.

This study contributes to our understanding of HRQoL in children by revealing the effect of health behaviour patterns on HRQoL. Where we used the EQ-5D-Y, other studies have used other measures such as the KIDSCREEN-10, the Pediatric Quality of Life Inventory (PedsQL 4.0) [17,19]. One advantage of the EQ-5D-Y is that it is simple and easy to complete by children. However, no studies have applied the EQ-5D-Y to investigate HRQoL in relation to health behaviour patterns. The findings in this study further support the reliability (Cronbach’s alpha = 0.61) and validity of the EQ-5D-Y among Canadian children.

This study has several strengths. We used a large, population-based, provincially representative sample of school children aged 10–11 years in Alberta, Canada. We used LCA, which is a model-based approach, to derive health behaviour patterns. This study assessed and analyzed a range of health behaviours, which helps facilitate the interpretation of results, and the comparison with other studies. Objective measures of student height and body weight were used in this study, which is more accurate than self-report. Several limitations should also be mentioned. The cross-sectional study design precludes making causal inferences regarding health behaviour patterns and health status. Longitudinal studies may help further elucidate how the behaviour patterns at baseline or changes in behaviour patterns over time influence future health outcomes. The survey was administered among children in Alberta, Canada, thus its findings should be generalized to other populations with caution. Future studies using data in other jurisdictions will facilitate comparisons and generalizability of the present findings. Lastly, this study relied on self-reported measures for health behaviours, which may be prone to recall or social desirability bias. While objective measures are desirable, it is usually not feasible to use such measures in large, population-based surveys.

Health interventions, policies, and programs that promote health of school-age children should give priority to targeting those subgroups with more unhealthy behaviours, and consider their co-existence or co-occurrence in relation to health status. These interventions, policies, and programs should be more effective by targeting health behaviours collectively rather than separately. Research has revealed that a comprehensive school health promotion approach (i.e., an approach that promotes health behaviours collectively) is most effective in enhancing the health of children and adolescents [25,43]. Additionally, interventions, policies, and programs should prioritize children and adolescents in socio-economically disadvantaged settings (e.g., lower family income), and with excess body weight (e.g., obesity). Further research should be prospective in design to solidify the causal link between health behaviour patterns and HRQoL. 

## 5. Conclusions

This study identified three distinct patterns of lifestyle behaviours and revealed unique associations of these patterns with HRQoL, socio-demographic characteristics, body weight, and diet quality. Children who undertook unhealthy lifestyle behaviours had lower HRQoL, and a higher likelihood of being overweight and obese. The findings suggest that health behaviours tend to exhibit heterogeneous response patterns and thus induce different effects on HRQoL; poorer behavioural patterns had greater negative effects on children’ health than healthier patterns. The findings suggest that health promotion strategies may be more effective when considering the patterns of health behaviours within populations as distinct targets in the efforts to improve HRQoL. Further research is warranted to better elucidate the relationship between health behaviour patterns and HRQoL by designing and executing prospective observational and intervention studies.

## Figures and Tables

**Table 1:** Socio-demographic characteristics and body weight status of grade five students participating in the 2014 REAL Kids Alberta survey (*n* = 2866).

	%
Gender	
Girls	52.88
Boys	47.12
Region of residence	
Urban	60.38
Rural	39.62
Parental education	
Secondary school or less	23.41
College	33.61
University	37.65
Missing	5.33
Household income (CAD/per year)	
≤50,000	13.67
50,001–75,000	8.58
75,001–100,000	10.57
>100,000	28.34
Do not know/prefer not to answer/missing	38.84
Body weight status	
Normal weight	68.41
Overweight	20.64
Obese	7.85
Missing	3.10

**Table 2:** Frequency distributions of students by health behaviours according to latent groupings, the 2014 REAL Kids Alberta survey.

	Group 1	Group 2	Group 3	Total
	*n* (%)	*n* (%)	*n* (%)	*n* (%)
Group size	1635 (54.76)	686 (24.04)	545 (21.20)	2866
Using a computer				
<1 h/day	1622 (99.76)	549 (79.78)	206 (35.93)	2377 (81.50)
1–2 h/day	4 (0.24)	68 (10.88)	206 (40.78)	278 (11.35)
≥3 h/day	0 (0)	58 (9.35)	127 (23.29)	185 (7.15)
Playing video games				
<1 h/day	1536 (94.64)	494 (73.89)	185 (36.88)	2215 (77.45)
1–2 h/day	83 (5.36)	79 (12.02)	144 (27.47)	306 (11.63)
≥3 h/day	0 (0)	102 (14.09)	206 (35.65)	308 (10.92)
Watching TV				
<1 h/day	1358 (84.58)	442 (64.74)	250 (47.15)	2050 (71.86)
1–2 h/day	201 (11.92)	112 (16.80)	121(22.14)	434 (15.26)
≥3 h/day	58 (3.50)	121 (18.46)	169 (30.71)	348 (12.88)
PA without a coach				
Never	194 (14.41)	90 (14.83)	175 (34.60)	459 (18.79)
1–3 times/week	745 (48.03)	252 (39.32)	256 (47.82)	1253 (45.90)
≥4 times/week	643 (37.56)	317 (45.85)	99 (17.58)	1059 (35.31)
PA with a coach				
Never	404 (27.24)	219 (35.33)	256 (50.69)	879 (34.16)
1–3 times per week	804 (50.63)	287 (42.51)	216 (39.49)	1307 (46.32)
≥4 times per week	365 (22.13)	148 (22.15)	56 (9.83)	569 (19.52)
Bed time on school days				
Before 9 p.m.	986 (61.41)	393 (57.69)	212 (38.30)	1591 (55.57)
9–10 p.m.	578 (38.12)	245 (38.84)	233 (43.73)	1056 (39.5)
After 10 p.m.	6 (0.47)	20 (3.46)	82 (17.98)	108 (4.93)
Bed time on weekends				
Before 9 p.m.	243 (15.07)	81 (11.22)	52 (8.55)	376 (12.75)
9–10 p.m.	754 (48.18)	312 (47.89)	154 (29.42)	1220 (44.10)
After 10 p.m.	568 (36.75)	263 (40.90)	318 (62.03)	1149 (43.15)
Vegetables and fruit				
≥5 servings/day	397 (24.73)	566 (83.32)	70 (14.30)	1033 (36.65)
<5 servings/day	1227 (75.27)	117 (16.68)	470 (85.70)	1814 (63.35)
Grain products				
≥5 servings/day	171 (10.67)	636 (93.87)	73 (13.23)	880 (31.26)
<5 servings/day	1453 (89.33)	47 (6.13)	467 (86.77)	1967 (68.74)
Milk and alternatives				
≥3 servings	564 (35.09)	542 (80.54)	180 (30.37)	1286 (45.05)
<3 servings/day	1060 (64.91)	141 (19.46)	360 (69.63)	1561 (54.95)
Meat and alternatives				
≥2 servings/day	63 (4.28)	527 (76.04)	39 (7.23)	629 (22.20)
<2 servings/day	1561 (95.72)	156 (23.96)	501 (92.77)	2218 (77.80)

PA: physical activity.

**Table 3:** The likelihood of reporting some or a lot of problems versus no problems for each of the EQ-5D-Y dimensions for the health behaviour patterns of grade five students participating in the 2014 REAL Kids Alberta survey.

	Walking	Looking after Myself	Doing Usual Activities	Having Pain or Discomfort	Feeling Worried, Sad or Unhappy
	OR (95% CI)	OR (95% CI)	OR (95% CI)	OR (95% CI)	OR (95% CI)
Health behaviour patterns					
Group 1	1.0	1.0	1.0	1.0	1.0
Group 2	1.33 (0.95, 1.86)	1.17 (0.89, 1.55)	**1.71 (1.31, 2.23)**	**1.41 (1.16, 1.71)**	**1.27 (1.01, 1.60)**
Group 3	**2.03 (1.48, 2.80)**	**1.59 (1.21, 2.09)**	**2.03 (1.50, 2.76)**	**1.50 (1.21, 1.85)**	**1.43 (1.08, 1.88)**
Gender					
Girls	1.0	1.0	1.0	1.0	1.0
Boys	1.08 (0.86, 1.36)	**0.78 (0.65, 0.95)**	**0.70 (0.57, 0.87)**	**0.77 (0.65, 0.92)**	**0.69 (0.58, 0.83)**
Region of residence					
Urban	1.0	1.0	1.0	1.0	1.0
Rural	1.22 (0.94, 1.60)	1.02 (0.79, 1.31)	1.05 (0.84, 1.31)	1.15 (0.93, 1.40)	**1.26 (1.00, 1.59)**
Parental education					
Secondary school or less	1.0	1.0	1.0	1.0	1.0
College	1.05 (0.75, 1.46)	1.18 (0.89, 1.56)	1.36 (0.97, 1.89)	0.98 (0.77, 1.25)	0.98 (0.78, 1.24)
University	0.77 (0.57, 1.03)	1.03 (0.76, 1.40)	1.00 (0.75, 1.33)	0.89 (0.71, 1.11)	0.93 (0.73, 1.17)
Household income, CAD					
≤50,000	1.0	1.0	1.0	1.0	1.0
50,001–75,000	**0.58 (0.35, 0.94)**	**0.59 (0.37, 0.96)**	0.78 (0.52, 1.18)	0.80 (0.54, 1.17)	0.75 (0.52, 1.07)
75,001–100,000	0.87 (0.56, 1.36)	**0.61 (0.40, 0.94)**	0.94 (0.60, 1.47)	0.80 (0.58, 1.09)	**0.70 (0.52, 0.95)**
>100,000	0.73 (0.48, 1.11)	**0.54 (0.37, 0.79)**	**0.69 (0.49, 0.99)**	0.95 (0.70 1.28)	0.76 (0.57, 1.01)

OR: odds ratio. CI: confidence interval. Notes: Regression models for each of the EQ-5D-Y dimensions are adjusted for gender, region of residence, parental education, and household income. Bolded *p*-values indicate statistical significance (*p* < 0.05).

**Table 4:** The likelihood of group membership according to socio-demographic characteristics, body weight status, and diet quality among grade five students participating in the 2014 REAL Kids Alberta survey.

	Group 2 versus Group 1	*p*-Value	Group 3 versus Group 1	*p*-Value
	OR (95% CI)		OR (95% CI)	
Gender				
Girls	1.0		1.0	
Boys	1.70 (1.36, 2.11)	**<0.001**	2.06 (1.66, 2.55)	**<0.001**
Region of residence				
Urban	1.0		1.0	
Rural	0.88 (0.67, 1.14)	0.333	0.46 (0.35, 0.60)	**<0.001**
Parental education				
Secondary school or less	1.0		1.0	
College	0.81 (0.65, 1.01)	0.059	0.69 (0.49, 0.95)	**0.023**
University	0.74 (0.55, 0.99)	**0.042**	0.87 (0.64, 1.19)	0.379
Household income, CAD				
≤50,000	1.0		1.0	
50,001–75,000	0.85 (0.55, 1.32)	0.481	1.00 (0.64, 1.56)	0.997
75,001–100,000	0.70 (0.45, 1.09)	0.114	0.52 (0.31, 0.87)	**0.013**
>100,000	0.75 (0.54, 1.03)	0.079	0.52 (0.37, 0.73)	**0.000**
Body weight status				
Normal weight	1.0		1.0	
Overweight	1.31 (1.04, 1.66)	**0.024**	1.44 (1.05, 1.97)	**0.023**
Obese	0.88 (0.56, 1.39)	0.586	1.41 (1.00, 1.98)	**0.050**
Diet quality				
Lowest tertile	1.0		1.0	
Middle tertile	7.13 (4.83, 10.52)	**<0.001**	0.85 (0.69, 1.05)	0.141
Highest tertile	11.85 (7.94, 17.69)	**<0.001**	0.68 (0.51, 0.89)	**0.006**

OR: odds ratio, CI: confidence interval. Group 1 was the reference group in the multinomial logistic regression. Bolded *p*-values indicate statistical significance (*p* < 0.05).

## Data Availability

Study data for the analyses were secondary data. The source datasets presented in this paper are not readily available due to privacy and confidentiality restrictions. Requests to access the datasets should be directed to P.J.V. (paul.veugelers@ualberta.ca).

## Supplementary Materials

The following supporting information can be downloaded at: https://www.mdpi.com/article/10.3390/children11030346/s1, Table S1. Measures used for the health behaviour patterns and their coding in the latent class analysis. Table S2. Fit statistics of the latent class models using 11 health behaviour items, grade five students participating in the 2014 REAL Kids Alberta survey (*n* = 2866). Table S3. Logistic regression for the association between the health behaviour patterns and overweight or obesity (*n* = 2763). Table S4. Socio-demographic characteristics, body weight status, diet quality, and the EQ-5D-Y dimensions of each health behaviour pattern group among grade five students participating in the 2014 REAL Kids Alberta survey. Table S5. Mean and standard deviation of the DQI-I score and four diet intake groups: vegetables and fruit, grain products, milk and alternatives, and meat and alternatives.
