# Supplementary material for: Health-Related Quality of Life in Relation to Health Behaviour Patterns among Canadian Children
Source: Children (Basel). 2024 Mar 14;11(3):346. doi: 10.3390/children11030346 (PMC10969172; doi:10.3390/children11030346)
Supplement: Supplementary file 1 [file children-11-00346-s001.zip › children-2876679-supplementary.pdf]

Supplementary information

Table S1. Variables used for the health behaviour classes and their coding in the latent class analysis

Variable	Response level	Analytic coding
Using a computer	Less than one hour/day<br>1-2 hours/day<br>≥ 3 hours/day	0<br>1<br>2
Playing video games	Less than one hour/day<br>1-2 hours/day<br>≥ 3 hours/day	0<br>1<br>2
Watching TV	Less than one hour/day<br>1-2 hours/day<br>≥ 3 hours/day	0<br>1<br>2
Physical activity with a coach	Never<br>1-3 times/week<br>≥ 4 times/week	2<br>1<br>0
Physical activity without a coach	Never<br>1-3 times/week<br>≥ 4 times/week	2<br>1<br>0
Bed time at weekdays	Before 9 pm<br>9-10 pm<br>After 10 pm	0<br>1<br>2
Bed time at weekend	Before 9 pm<br>9-10 pm<br>After 10 pm	0<br>1<br>2
Fruit and vegetables intake	Number of daily servings	0 = ≥ 5 daily servings<br>1 = < 5 daily servings
Grain products	Number of daily servings	0 = ≥ 5 daily servings<br>1 = < 5 daily servings
Milk and alternatives	Number of daily servings	0 = ≥ 3 daily servings<br>1 = < 3 daily servings
Meat and alternatives	Number of daily servings	0 = ≥ 2 daily servings<br>1 = < 2 daily servings

Table S2. Fit statistics of the latent class models using 11 health behaviour items, grade five children participating in the 2014 Real Kids Alberta survey, Canada (n = 2866)

Classes	# Par	BIC	aBIC	LMRALRT p-value	Class proportions			
					Class 1	Class 2	Class 3	Class 4
1-class	18	47723.1	47665.9	NA	1.00			
2-class	37	46085.8	45968.1	< 0.0001	0.71	0.29		
**3-class**	**56**	**45430.0**	**45252.1**	**0.0096**	**0.55**	**0.24**	**0.21**	
4-class	75	45072.5	44834.2	0.3226	0.37	0.31	0.21	0.11

Par: number of free estimated parameters; BIC: Bayesian information criterion; aBIC: sample size adjusted BIC; LMRALRT: Lo-Mendell-Rubin adjusted likelihood ratio test. The class proportions were based on the classification of individuals based on their most likely latent class membership. The latent class analyses were weighted to accommodate the design effect such that the estimates represent the population of grade five children in the province of Alberta.

Table S3. Logistic regression for the association between the health behaviour patterns and overweight or obesity (n = 2763)

**Variables**	**Overweight and obese versus normal weight**
	**OR (95% CI)**
**Health behaviour patterns** (reference: Class 1)	
Class 2	1.18 (0.92, 1.51)
Class 3	**1.32 (1.01, 1.72)**
**Gender** (reference: Girls)	
Boys	1.12 (0.94, 1.34)
**Residence (**reference: Urban)	
Rural	0.89 (0.70, 1.13)
**Parental education (ref.:** Secondary school or lower)	
College	0.92 (0.73, 1.15)
University or above	0.93 (0.73, 1.18)
**Household income** (ref.: ≤ $50,000)	
$50,001-$75,000	0.96 (0.65, 1.41)
$75,001-$100,000	0.74 (0.51, 1.08)
> $100,000	**0.56 (0.41, 0.76)**

Bold values indicate a statistically significance ($p < 0.05$).

Table S4. Within class frequency distribution (%) by the socio-demographics, body weight, diet quality and the EQ-5D-Y dimensions, grade five students participating in the 2014 Real Kids Alberta survey in Canada

**Variables**	**Class 1**	**Class 2**	**Class 3**
**Gender** (n = 2865)			
Girls	59.06	46.97	42.22
Boys	40.40	53.03	57.78
**Residence** (n = 2866)			
Urban	56.43	58.35	72.80
Rural	43.57	41.65	27.20
**Parental education** (n = 2866)			
Secondary school or lower	21.32	25.92	25.91
College	35.10	34.23	29.02
University or above	38.51	33.90	39.74
Missing	5.06	5.95	5.32
**Household income** ($CA/per year) (n = 2866)			
≤ $50,000	11.32	15.64	17.50
$50,001-$75,000	7.53	8.59	11.24
$75,001-$100,000	11.46	10.33	8.51
> $100,000	30.72	28.60	21.99
Do not know/not to answer/missing	38.96	36.83	40.76
**Body weight status** (n = 2866)			
Normal weight	71.22	66.68	63.02
Overweight	18.36	22.97	23.84
Obese	7.44	6.74	10.18
Missing	2.98	3.61	2.96
**DQI-I** (n = 2847)			
Lowest tertile	40.09	6.74	46.94
Middle tertile	31.51	36.81	30.66
Highest tertile	28.40	56.45	22.39
**EQ-5D-Y dimensions**			
**Walking** (n = 2850)			
No problems	89.31	86.15	80.81
Some or a lot of problems	10.69	13.85	19.19
**Looking after self** (n = 2843)			
No problems	85.65	83.55	79.23
Some or a lot of problems	14.35	16.45	20.77
**Doing usual activities** (n = 2843)			
No problems	87.87	81.42	79.37
Some or a lot of problems	12.13	18.58	20.63
**Having pain or discomfort** (n = 2848)			
No problems	58.75	51.16	50.41
Some or a lot of problems	41.25	48.84	49.59
**Feeling worried, sad or unhappy** (n = 2847)			

No problems	73.04	68.81	67.27
Some or a lot of problems	26.96	31.19	32.73

Weighted percentages are shown in the table.

Table S5. Mean and standard deviation of the DQI-I score and four diet intake groups: vegetables and fruit, grain products, milk and alternatives, and meat and alternatives (n = 2847)

**Variables**	**Mean**	**SD**
**Overall**		
Fruit and vegetables (servings/day)	4.93	3.74
Grain products (servings/day)	4.52	2.85
Milk and alternatives (servings/day)	3.21	2.17
Meat and alternatives (servings/day)	1.51	1.01
DQI-I score	61.80	10.31
**By diet intake category levels**		
Vegetables and fruit		
≥5 servings/day	8.72	3.68
<5 servings/day	2.77	1.17
Grain products		
≥5 servings/day	7.60	3.16
<5 servings/day	3.14	1.07
Milk and alternatives		
≥3 servings/day	5.09	1.83
<3 servings/day	1.67	0.73
Meat and alternatives		
≥2 servings/day	2.97	1.06
<2 servings/day	1.10	0.47

SD: standard deviation of the mean value
